# Complete genome sequences of two *Mesoterricola* strains belonging to the phylum *Acidobacteriota*, isolated from sediment and paddy soil

**DOI:** 10.1128/mra.01397-25

**Published:** 2026-03-12

**Authors:** Reika Isoda, Miyu Kuniyasu, Hideomi Itoh

**Affiliations:** 1 Biomanufacturing Process Research Center, National Institute of Advanced Industrial Science and Technology 13508, Sapporo, Hokkaido, Japan; 2 Department of Biotechnology, Hokkaido High-technology College, Eniwa, Hokkaido, Japan; University of Southern California 5116, Los Angeles, California, USA

**Keywords:** *Acidobacteriota*, genome, anaerobe, *Mesoterricola*, soil

## Abstract

*Mesoterricola* strains MK458 and JemC181 are anaerobic bacteria within the phylum *Acidobacteriota*, isolated from sediment and paddy soil, respectively. Here, we report their complete genomes, ranging from 4.76 to 5.18 Mb in size, with G + C contents of 68.3%–68.6%, encoding 4,021–4,435 protein-coding genes.

## ANNOUNCEMENT

*Mesoterricola* spp., first isolated in 2023, are facultatively anaerobic bacteria of the phylum *Acidobacteriota* inhabiting soil environments (1). The cells are motile rods capable of reducing Fe(III) compounds as electron acceptors (1). Here, we report the complete genome sequences of two *Mesoterricola* strains, MK458 and JemC181.

MK458 was previously isolated from river sediment (43°00′43.7″N, 141°42′12.8″E) using the soil slurry method (1), and JemC181 was newly isolated from paddy soil (37°26′15.7″N, 138°52′19.0″E) using the same procedure. Briefly, for both strains, fresh soil samples, collected (0–3 cm depth, ~20 g) with a sterile spatula and stored at 4°C, were added to a slurry containing autoclaved soil and incubated anaerobically, followed by single-colony isolation on 1.5% R2Af agar plates (2) under anaerobic conditions using AnaeroPack sachets (Mitsubishi Gas Chemical, Japan) at 30°C. Both strains were cultured for one week for DNA extraction. DNA from MK458 was extracted as described previously (3), and the same DNA preparation was used for all sequencing libraries, whereas JemC181 cells were frozen in liquid nitrogen, ground, and processed using a Genomic-tip 20/G kit (Qiagen, Germany). Default parameters were used for all software unless otherwise specified.

For MK458, DNA sequencing combined short- and long-read technologies using DNBSEQ-G400 (BGI, China) and GridION X5 (Oxford Nanopore, UK). Short-read libraries were prepared from 129 ng DNA with 10 amplification cycles using the MGIEasy FS DNA Library Prep Set, circularized with the MGIEasy Circularization Kit, and sequenced (2 × 200 bp). Long-read libraries were prepared from 1,000 ng DNA using the Ligation Sequencing Kit (SQK-LSK109), without size selection, and sequenced on a GridION with R9.4.1 flow cells and Guppy v4.0.11+f1071 ce basecaller (N_50_ 5,897 bp). Short-read adapters were trimmed with Cutadapt v2.7 (4); low-quality reads (Q < 20) and reads <127 bp were removed with Sickle v1.33 (5). Long-read adapters were removed with Porechop v0.2.3 (https://github.com/rrwick/Porechop), and reads <1,000 bp were filtered with Filtlong v0.2.0 (6). Hybrid assembly based on 5,474,814 short and 203,804 long reads was performed with Unicycler v0.4.7 (7). For JemC181, sequencing was performed on the PacBio Revio (PacBio, USA). DNA was purified with 0.88× ProNex beads, fragmented to 10–20 kbp using g-TUBE (Covaris), and 2,000 ng DNA was used for library preparation with the SMRTbell Express Template Prep Kit 2.0. Reads with Q < 20 or <1,000 bp were removed with SMRT Link v12.0.0 and Filtlong v0.2.1 (6) (30,143 HiFi reads, N_50_ 9,343 bp) and assembled with Flye v2.9.2 (8). Circularity, quality, and annotation were evaluated using Bandage v0.8.1 (9), CheckM2 v1.0.1 (10), and DFAST v1.6.0 (11), without reorientation. Average nucleotide identity (ANI) was calculated using JSpeciesWS v5.0.2 (12).

The complete genomes of MK458 (4.76 Mb; 454× coverage; G + C content 68.3%; completeness 99.1%; contamination 1.8%) and JemC181 (5.18 Mb; 48× coverage; G + C content 68.6%; completeness 98.8%; contamination 2.1%) encode 4,021 and 4,435 protein-coding genes and 57 and 50 tRNAs, respectively, and 2 rRNA operons. ANI values between MK458 and JemC181 (88.5%) and to *M. silvestris* W79^T^ (86.5% and 87.4%, respectively) indicate that both strains likely represent distinct species (13).

## Data Availability

The partial 16S rRNA gene sequences of strain MK458 is available in the DDBJ/ENA/GenBank under accession number OP984662. The complete genome sequences of strains MK458 and JemC181 are available in the DDBJ/ENA/GenBank under accession numbers AP045075 and AP045076, respectively, as part of BioProject PRJDB38050. The corresponding raw sequencing reads are available in the DDBJ Sequence Read Archive under accession numbers DRR888679 and DRR888680 (for MK458), and DRR888681 (for JemC181).
